# The Dual Role of Microglia in Multiple Sclerosis and its Implications for Diagnostics and Repair

**DOI:** 10.2174/011570159X352356241126044933

**Published:** 2025-03-07

**Authors:** Melissa T. Goulart, Davi T.U. Queiroz, Fabíola M. Ribeiro

**Affiliations:** 1 Department of Biochemistry and Immunology, Institute of Biological Sciences, Universidade Federal de Minas Gerais, Belo Horizonte, Brazil;; 2 Department of Neurology, Felício Rocho Hospital, Belo Horizonte, Brazil

**Keywords:** Multiple sclerosis, microglia, oligodendrocytes, demyelination, neuroinflammation, disease-modifying drugs

## Abstract

Microglia play a crucial role in the development, immune surveillance, and repair of the central nervous system. These cells play a multifaceted role in multiple sclerosis (MS), with evidence suggesting that microglia can promote both active inflammation and remyelination. For instance, it has been shown that microglia can support the development of oligodendrocytes and phagocytose myelin debris, thus aiding in proper remyelination. However, microglia overactivation in MS lesions exacerbates neuroinflammation by releasing inflammatory cytokines and facilitating the activation of astrocytes and immune cells, promoting demyelination and, ultimately, driving MS pathology. In fact, it has been shown that there is a correlation between activated microglia patterns and the chronicity of MS. Thus, although it is difficult to be certain whether these cells are friends or foes, there is no doubt that microglia will be a relevant target for MS diagnosis and treatment in the future, when further research will help to clarify the role of these cells in MS. MRI and PET scan allow evaluation of microglia/macrophages biomarkers, facilitating the clinical assessment of a patient's disease stage. Moreover, new microglia-specific markers are being discovered, which will increase diagnostic precision, helping to identify active and chronic MS lesions. Because microglia are involved in all MS phases, these cells are also an important drug target. In this review, we focus on the current understanding of the role of microglia in MS progression as well as on the evidence supporting both inflammatory and reparative functions of these cells. We will also review how microglia may yield new biomarkers for MS diagnosis and serve as a potential target for therapy.

## 1 INTRODUCTION

### 1.1 Microglia Origin and Functions in the Healthy and Diseased Brain

Microglia are mesodermal cells derived from the yolk sac, differing from other glial cells, as oligodendrocytes and astrocytes, which originate from the neuroectoderm [1]. Initially thought to derive from macrophages and tissue monocytes, it is now established that microglia originate from mesodermal progenitor cells that colonize the central nervous system (CNS) early during embryogenesis. These cells enter the CNS through the leptomeninges and lateral ventricles, spreading throughout the cerebral cortex, and being maintained over time by endogenous cells and circulating monocytes [2, 3]. Just as macrophages, which are primarily regulated by colony-stimulating factor 1 (CSF-1), microglial development is also highly dependent on the colony-stimulating factor 1 receptor (CSF-1R) and its ligand, CSF-1. In homozygous mice with a null mutation in the Csf1r gene, microglia are depleted by more than 99% during the embryonic stage, and, after birth, microglial disruption persists in most brain regions, leading to structural modifications in the brain [4]. Moreover, transforming growth factor (TGF)-β1 signaling is essential for microglia maturation and homeostasis, as well as for preventing microglial excessive activity [5]. Human microglia are very similar to mouse microglia, although certain genes are expressed in greater quantities in the human brain, including genes encoding complement system molecules, such as C3 and C2, as well as transcription-regulating genes [6]. Both human and mouse microglia respond to inflammatory contexts or injuries, indicating a conserved degree of cellular mechanisms and functions [7]. Therefore, studies using animal models can provide important insights.

To understand how microglia function in disease contexts, it is necessary to first comprehend their roles in a healthy environment. In a healthy CNS, microglia represent about 10% of resident cells, although this percentage can vary by region, ranging from 5% to 12% [8]. They are considered self-renewing cells and participate in tissue remodeling of the brain and spinal cord throughout the maturation of these tissues. In the healthy CNS, they act as the main immune cells, functioning similarly to peripheral macrophages [9]. Under homeostatic conditions, they surveil the CNS, investigating potential damage and acting to prevent exacerbated responses. Microglia produce neurotrophic factors, such as insulin-like growth factor-1 (IGF-1), which promotes the maturation of oligodendrocyte precursor cells and trigger myelination [10], and brain-derived neurotrophic factor (BDNF), which plays a crucial role in neuronal function and also facilitates myelination [11-13]. In line with the idea that there are various subpopulations of microglia exerting specific roles, it has been proposed that CD11c^+^ microglia are responsible for producing IGF-1, facilitating myelination and neurogenesis in the developing mouse brain [12]. Moreover, during fetal development, microglia have been shown to be important for guiding neuronal migration, pruning synaptic terminals, and promoting myelin formation [14].

Microglia can orchestrate immune responses to infection and tissue damage, balancing tissue reaction and recovery [15]. Their actions can be triggered by various molecules, such as cytokines, chemokines, hormones, neurotransmitters, and adhesion molecules, and they can sense and process these signals through either microglial receptors or indirectly, through other cells [16]. Microglial defense activity can be essential in neurodegenerative diseases by clearing harmful and aggregating proteins, such as α-synuclein, mutant huntingtin, β-amyloid, prions, and oxidized or mutant superoxide dismutase [15]. This response can be initiated through Toll-like receptors (TLRs), leading to neuroinflammatory responses and cytokine release, ultimately aiding in clearing these proteins [17].

Upon detecting environmental changes, microglia can morphologically modify their processes. These modifications are highly dynamic, allowing their branches to sense areas around the cell body and polarize toward potential injuries [18]. Analysis of microglia morphological alterations can be helpful to understand biological processes involving these cells in various contexts, including neurodegenerative diseases [19]. Microglia can be subdivided based on morphology, cell surface receptors, molecule secretion, and functional properties [20]. In neurodegenerative diseases, such as AD, Parkinson’s disease, Amyotrophic Lateral Sclerosis (ALS), and multiple sclerosis (MS), microglia can assume various phenotypes [21]. Until recently, microglia were dichotomously classified as M1 and M2, with M1 representing classically activated neurotoxic cells and M2 representing alternatively activated neuroprotective cells [22]. This classification was also applied to other CNS cells, including astrocytes, which were subdivided into A1 and A2 [23]. However, this dichotomy is overly simplistic and inaccurate, as the cellular profiles are much more diverse, as revealed by new techniques, including RNA sequencing, which showed the temporal and spatial diversity of microglia cells [1, 24, 25]. Additionally, microglial gene expression varies across CNS tissues, highlighting the complexity of microglia activation. For instance, gray matter microglia exhibit increased expression of type 1 interferon (IFN)-related genes and complement genes, whereas white matter microglia show higher expression of nuclear factor-κ B (NF-κB) inhibitory genes [26].

Upon detecting damage, microglia undergo transformations in gene expression, metabolism, motility, morphology, and proliferation. Morphological changes in processes begin within minutes of detecting CNS damage, with processes retracting over days and microglia assuming amoeboid characteristics, typical of macrophages [27]. However, in response to stress or aging, they can become hyper-ramified [28, 29]. Morphological changes alone do not characterize microglial activation, as a rapid response to CNS damage is mainly due to the high expression of damage-associated molecular pattern (DAMP) and pathogen-associated molecular pattern (PAMP) receptors on their surface. Consequently, microglia are essential for initiating inflammatory responses in infectious, neuroinflammatory, and neurodegenerative contexts [1, 24]. Microglial activation can lead to a series of consequences, both positive and negative. Microglia's detrimental role encompasses the release of pro-inflammatory cytokines, such as IL-1β, reactive oxygen species, nitric oxide, and proteases. On the other hand, microglia's beneficial functions include the release of anti-inflammatory cytokines, such as IL-10, and growth factors, including TGF-β, as well as the promotion of tissue repair, reconstruction of extracellular matrix, and phagocytosis of apoptotic cells [22]. The paradoxical role of microglia activation is well exemplified in Alzheimer's disease (AD), where disease-associated microglia (DAM) were identified near Aβ plaques, being responsible for clearing β-amyloid [30], although activated microglia was also shown to increase neuroinflammation and phosphorylate Tau protein [31]. Although the effects can range between two opposite poles, good or bad, it is worth noting that the understanding of what can be beneficial or harmful is still evolving. This can be exemplified in situations where a cytokine typically characterized as either pro-inflammatory or anti-inflammatory may act in the opposite way to its conventional role. For example, the cytokine IL-12, traditionally characterized as a pro-inflammatory cytokine, in an inflammatory and autoimmune context, such as the EAE model, can act to limit neuroinflammation [32]. As in several neurodegenerative diseases, microglia participate in many processes in the context of MS.

## 2 MULTIPLE SCLEROSIS

MS is an autoimmune, chronic, demyelinating neurological disease, most commonly occurring in young adults. MS prevalence is higher in women, with a ratio of 3:1. Incidence peaks at 29 years of age for women and 31 years for men, with a subsequent decrease in incidence above 40 years of age for both sexes [33]. The etiology of MS is multifactorial, involving genetic factors related to the human leukocyte antigen (HLA) gene as both risk and protective factors, as well as environmental factors, such as lifestyle habits, including increased body mass index, smoking, and infections, such as Epstein-Barr virus infection [34]. Although the pathophysiological process of MS still has some loose ends, it is well known that in the early stages of the disease, the rupture of the blood-brain barrier (BBB) facilitates the infiltration and accumulation of CD8^+^ T lymphocytes and monocyte-derived macrophages, primarily around post-capillary venules in the periventricular white matter and in the cortex, which are key processes in the development of multiple sclerosis lesions [35]. T cell activation plays a crucial role in the immunopathogenesis of MS. Helper T cells (Th cells), particularly Th1 and Th17 cells, infiltrate the CNS and interact with microglia. Th1 cells, producing interferon-gamma (IFN-γ), and Th17 cells, secreting interleukin-17 (IL-17), are particularly important in mediating the inflammatory response and perpetuating tissue damage [35]. More recently, a subset of T helper cells known as ThGM, which produce granulocyte-macrophage colony-stimulating factor (GM-CSF), has also been shown to play a critical role in microglial activation and the amplification of inflammation within the CNS [36]. Meanwhile, B lymphocytes accumulate in clusters of ectopic lymphoid tissues in the meningeal region, mainly subpial, and in the perivascular space of blood vessels. They contribute to disease progression through multiple mechanisms, including antibody production, antigen presentation, and the secretion of pro-inflammatory cytokines [37]. These B cell-rich areas have been associated with cortical demyelination and gray matter atrophy, contributing to the disease's clinical progression [35, 38, 39]. B cells act as potent antigen-presenting cells, providing costimulatory signals to T cells and promoting their activation. This interaction between B cells and T cells further intensifies the inflammatory cascade within the CNS. Additionally, B cells secrete cytokines such as interleukin-6 (IL-6) and tumor necrosis factor-alpha (TNF-α), which promote the activation of microglia. Peripheral cells join the resident cells of the CNS, astrocytes, and microglia, which are now activated and, together, they promote demyelination and lesions to oligodendrocytes and neurons. This process occurs through direct cell-cell contact or *via* mechanisms dependent on the secretion of inflammatory and neurotoxic mediators [40].

**Fig. (1):**
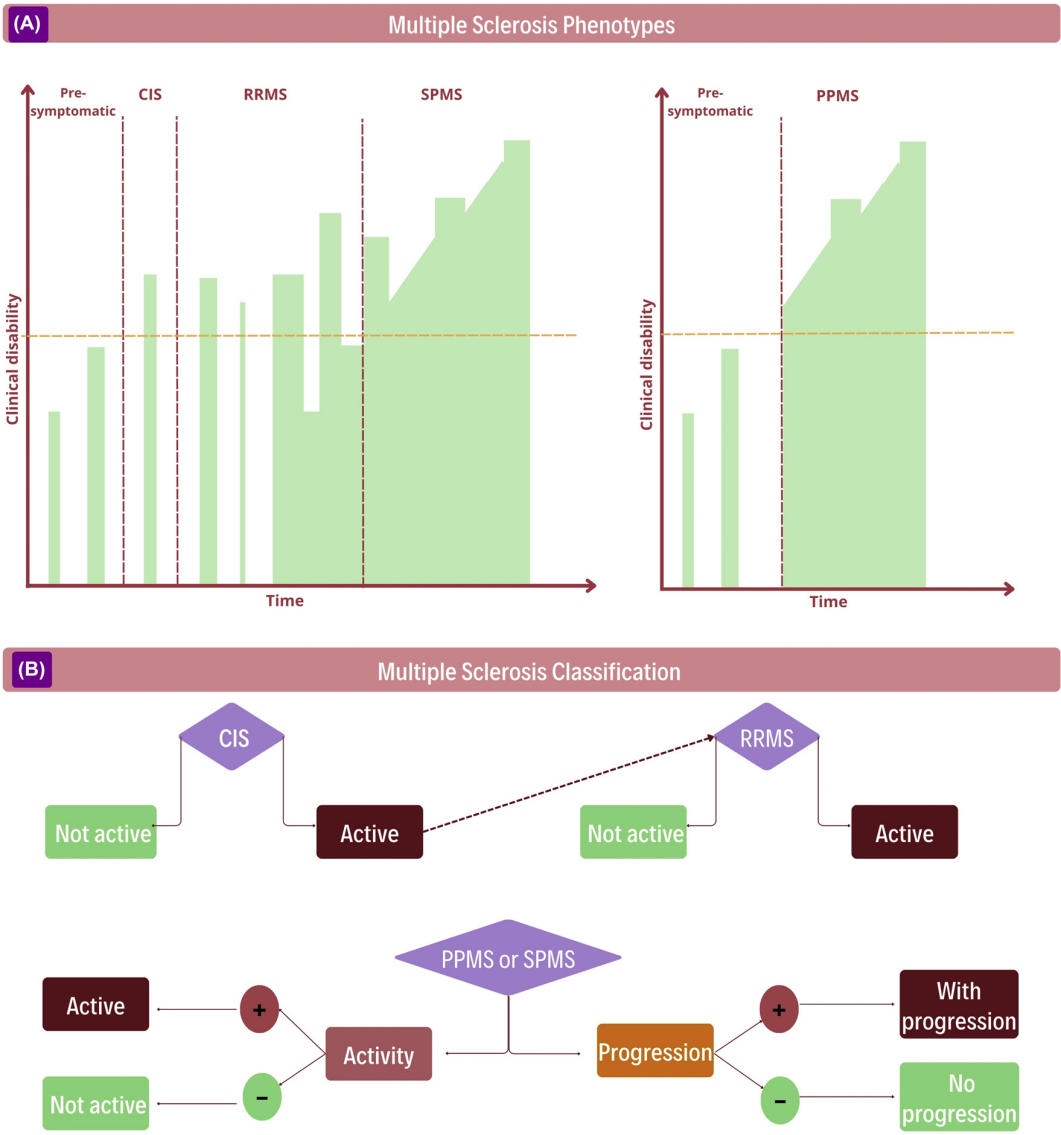
(**A**) Graphs show the clinical progression of disability accumulation over time for different MS phenotypes. The left graph represents the RRMS phenotype, marked by relapses, which evolves into the SPMS form that may present relapses, but with a predominance of PIRA. It is important to note that there may be disease activity below a clinical threshold (dashed yellow line), which may correspond, for example, to changes in magnetic resonance imaging. The right graph represents the PPMS form, where PIRA predominates from the beginning, but relapses can also occur during disease progression. (**B**) Illustrative scheme of multiple sclerosis (MS) phenotypes according to Lublin *et al*. 2013. **Abbreviations**: CIS: clinically isolated syndrome; RRMS: relapsing-remitting MS; SPMS: secondary-progressive MS; PPMS: primary progressive MS; PIRA: progression independent of relapse activity.

The disease course comprises different phenotypes. Following its 2013 revision, Lublin *et al*. described its subdivision into clinically isolated syndrome (CIS), with patients exhibiting typical clinical symptoms of MS with correlating magnetic resonance imaging findings, but without meeting the McDonald clinical criteria for MS. The relapsing-remitting form (RRMS), characterized by recurrent attacks that may or may not be followed by a full recovery, is the most typical, affecting approximately 85% of MS patients. Of these, 50% of patients experience symptom deterioration independent of relapses (progression independent of relapse activity - PIRA), categorizing them as having the secondary progressive form (SPMS). Moreover, 15% of all patients present with progressive worsening of neurological symptoms from onset, classified as primary progressive MS (PPMS). Even within progressive forms, attacks may persist or cease, leading to a subdivision into active or non-active forms (Figs. **1A** and **1B**) [41].

During relapses, patients exhibit characteristic neurological signs of central nervous system involvement, such as low visual acuity and dyschromatopsia (optic neuritis), sensory alterations, gait instability, and vertigo, among others. The spinal cord, brainstem, and optic nerve are most commonly affected [33, 42]. To be qualified as a relapse, symptoms must exhibit subacute onset, worsening over days until reaching a plateau. Additionally, infection or fever should be ruled out as confounding factors for clinical worsening (pseudo relapses) [43]. PIRA marks the onset of clinical progression phases, either in PPMS or SPMS, and manifests as persistent disability, worsening over 6 to 12 months without concurrent relapses during the evaluation period and within the three months prior to the onset of worsening [44]. Of note, patients with RRMS whose disease onset takes place after 40 years of age, are male, and experience spinal attacks tend to show signs of progression earlier than others [33, 45]. On average, such symptoms emerge approximately 15 years after MS onset. Disability can be assessed using the Expanded Disability Status Scale (EDSS), although limitations exist due to inter and intra-examiner reproducibility variation. Other assessments aiding disability determination include the nine-hole peg test (9HPT) and the time to 25-foot walk test (time to 25FWT), evaluating upper limb motor coordination and gait capacity, respectively. Attacks with incomplete recovery (relapses and worsening - RAW) contribute to performance deterioration in these tests over 10 years, serving as contributing factors for long-term disability accumulation. Additionally, cognitive function can be briefly assessed using the symbol-digit test (SDT), an important tool to evaluate patients with progressive forms of MS. PIRA also plays a crucial role in disability accumulation, especially in patients not receiving disease-modifying treatment [46].

## 3 DIFFERENT PHASES OF MULTIPLE SCLEROSIS

**Fig. (2):**
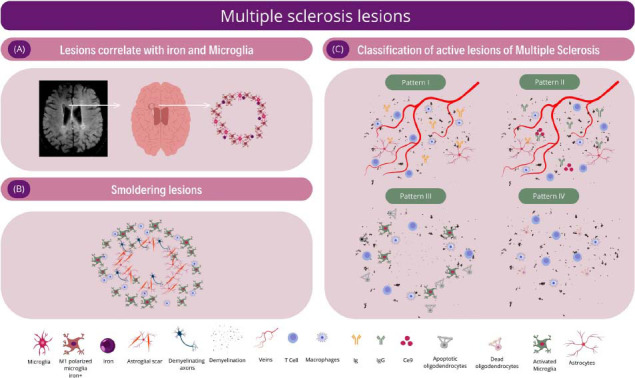
Panel of microglia-related lesions in multiple sclerosis. (**A**) MRI of a brain with a chronically active lesion and a border rich in activated microglia showing an axial brain slice in a magnetic susceptibility sequence, with the white arrow pointing to a ring-like hypointense region in the periventricular area on the right. This hypointense finding in susceptibility sequences corresponds to changes related to iron deposits in areas of multiple sclerosis (MS) lesions. There is a correlation between this finding and the histopathological analysis of chronically active lesions, which have a hypocellular inner region with a border rich in activated microglia. The presence of iron-laden microglia indicates chronic activation and ongoing neuroinflammation. In the right panel, representation of a lesion showing a ring of microglia with the presence of iron. (MRI provided, courtesy of Dr. Thyago Oliveira de Queiroz, neuroradiologist at Felício Rocho Hospital) (**B**) Smoldering lesions - The center of the lesion is inactive, with no remyelination or myelin proteins, and exhibits extensive axonal loss. There is the presence of astrogliotic scar tissue surrounding the demyelinated axons. At the edge of the lesion, activated microglia and macrophages appear, which may contain myelin degradation products. Additionally, there can be extensive and diffuse microglial activation in areas surrounding normal-appearing white matter. (**C**) Patterns of demyelinating lesions based on Lucchinetti *et al*., 2000. Pattern I: lesions marked by active demyelination associated with inflammation predominantly by T lymphocytes and macrophages. Additionally, there is diffuse immunoglobulin (Ig) reactivity in astrocytes. The demyelination plaques are centered on small veins and venules, with extensive loss of myelin proteins that form the myelin sheath. Pattern II: contains all the characteristics of Pattern I lesions but also has prominent IgG deposition and C9neo complement antigen. This marked Ig reactivity is associated with myelin degeneration. Pattern III: lesions with inflammatory infiltrates of T lymphocytes, macrophages, and activated microglia, characterized by significant loss of oligodendrocytes at the active plaque edge. The lesion center is inactive, devoid of oligodendrocytes, and lacks remyelination. Oligodendrocyte death is characteristic of apoptosis. Pattern IV: inflammatory infiltrate dominated by T lymphocytes and macrophages, where demyelination is associated with non-apoptotic oligodendrocyte death. There is almost complete loss of oligodendrocytes in both active and inactive areas of these lesions. Patterns I and II resemble autoimmune pathology, whereas Patterns III and IV are indicative of primary oligodendrocyte lesions, suggesting pathologies induced by non-autoimmune pathways.

The phenotypic presentation of MS, characterized by relapses or PIRA, closely correlates with the pathophysiological process of neuronal damage. Active/inflammatory lesions, predominant in initial disease phases, feature the involvement of T lymphocytes, with microglia playing a key role in myelin protein phagocytosis, antigen presentation to T lymphocytes, and release of proinflammatory cytokines [25]. Histopathological analysis of active MS lesions reveals a diffuse infiltrate of microglia, macrophages, T lymphocytes, and plasma cells [47]. These four components are also present during the demyelination phase, as well as post-demyelination, differing on the type of products found within microglia cytoplasm. Engulfment by microglia of minor myelin proteins, such as myelin oligodendrocyte glycoprotein (MOG), 2',3'-cyclic-nucleotide 3'-phosphodiesterase (CNP), and myelin-associated glycoprotein (MAG), along with major myelin proteins, including myelin basic protein (MBP) and proteolipid protein (PLP), characterize recent demyelinating lesions. Later demyelinating lesions entail the presence of major myelin proteins in microglia cytoplasm [48]. Active lesions can be classified into four patterns, according to Lucchinetti *et al*. classification. The four patterns of alterations in MS demonstrate distinct features. Patterns I and II share several similarities, with active demyelination characterized by T-lymphocyte and macrophage-dominated inflammation. The primary difference is the prominent deposition of immunoglobulins (mainly IgG) and complement C9neo antigen at sites of active myelin destruction in pattern II lesions, a feature absent from pattern I. Pattern III lesions differ as demyelination is not centered around veins and venules. Instead, these lesions frequently show a rim of preserved myelin around inflamed vessels within the demyelinated plaque. Pattern IV lesions also have inflammatory infiltrates dominated by T lymphocytes and macrophages, but lack deposition of immunoglobulins and complement C9neo antigen. Demyelination in these lesions is associated with oligodendrocyte death in a small rim of periplaque white matter adjacent to the zone of active myelin destruction (Figs. **2A**-**C**) [49]. During the progressive phase of MS, more pronounced in late disease stages, mixed lesions become common, characterized by chronic active lesions or “slowly expanding lesions” (“smoldering lesions”). These lesions feature a hypocellular center and significant activation of microglia and macrophages at their periphery (Fig. **2B**) [25, 50]. Iron deposition within the cytoplasm of activated microglia and macrophages occurs during this process, an uncommon finding in inactive lesions and absent during the remyelination process of active lesions, serving as a hallmark of chronic active lesion pathophysiology. Clinically, these findings correlate with a poorer prognosis and disability accumulation. Luchetti *et al*.'s study histologically evaluated brains from the Netherlands Brain Bank (NBB), demonstrating a positive correlation between acute lesions and microglial activation at the lesion site. Progressive phenotypes exhibit a higher proportion of chronic active lesions, directly associated with increased lesion load and reduced remyelinated lesion proportion. Moreover, the higher the proportion of chronic active lesions, the higher the patient's disability score (EDSS) at the time of death [51].

Given the importance of preventing disability accumulation and altering the natural history of MS, the early use of highly effective disease-modifying drugs has been a recurring topic of discussion. Consequently, various therapeutic targets have been studied. Microglia play a pivotal role in MS activity, both in early/inflammatory phases and during progression [52]. However, a drug mechanism with direct action on these cells resulting in a significant effect remains undiscovered, with current interventions exerting only indirect effects. The following topics in this review aim to comprehensively describe the multifaceted roles of microglia in MS, including their involvement in myelin damage and the processes of remyelination. Moreover, current methodologies to evaluate microglial activity in clinical practice and to identify potential therapeutic targets to modulate their function, aiming at improving treatment outcomes, will also be explored.

## 4 INTERACTION BETWEEN MICROGLIA AND OTHER CNS CELLS IN THE CONTEXT OF MS

Microglia are involved in various processes in MS, from disease onset to more advanced and chronic stages. The major debate is whether microglia plays a beneficial role or is merely a villain. It appears that the roles of microglia are ambiguous and highly dependent on the disease stage. As the primary immune cell of the CNS, microglia can communicate with various cell types, leading to a series of positive or negative actions, depending on the context [53, 54]. Astrocytes, microglia, and oligodendrocytes create a highly controlled and suitable environment in the CNS for neuronal and cellular survival in general [55]. They not only maintain neuronal homeostasis but also interact directly with other cells to promote a balanced response.

Microglial communication with almost all cell types can promote either the maintenance of homeostasis or the development of pathogenic cascades, which are common in neurodegenerative and inflammatory diseases such as MS [18]. Besides interacting with other glial cells, such as oligodendrocytes and astrocytes, a highly studied and extremely important interaction is with neurons. In homeostasis, the interaction between microglia and neurons is essential for the proper functioning of the CNS, as it can control neuronal survival, participate in neurogenesis, as well as form or prune synapses [56, 57]. Microglia can act on neurons by activating many pathways and secreting various molecules and signals, which can lead to several cascades of reactions [19]. Microglia-neuron communication is bidirectional, as the ability to control function and the activation of cell signaling pathways is not limited to microglia, with neurons also being able to trigger various microglial responses. Ronzano *et al*. (2021) showed that microglial interaction with the nodes of Ranvier is mediated by neurons, which leads to a pro-remyelination effect after myelin injury [58]. Additionally, the interaction of CD200 and its neuronal receptor can modulate microglial activation and regulate synaptic plasticity [59, 60]. Furthermore, the activation of NMDA receptors expressed by neurons can lead to microglial process branching [61]. In the experimental autoimmune encephalomyelitis (EAE) model, a classic experimental model of MS, microglial activation can significantly impair neuronal function by either releasing pro-inflammatory mediators that alter glutamatergic signaling or producing other factors such as reactive oxygen species, which can lead to synaptic and even cognitive alterations [22, 62, 63]. Conversely, some already approved and used disease-modifying treatments (DMTs) for MS can improve these processes by either reducing synaptic dysfunction in EAE or decreasing the release of inflammatory mediators by microglia [64-67].

Microglial communication with astrocytes begins early during neurodevelopment and CNS cell colonization, playing an important role in maintaining homeostasis [23]. In addition, this bilateral communication is essential for the neuroinflammatory processes that take place in MS [68]. This interaction occurs through the secretion of various signaling molecules, cytokines, chemokines, and extracellular vesicles. The consequences in MS can be beneficial or harmful, depending on the context. Microglia can secrete NF-κB, complement component 1q (C1q), IL-1α, and TNF-α, which can lead to a wide range of detrimental responses in the context of MS, including the induction of a pro-inflammatory phenotype in astrocytes [68]. These responses can range from activating inflammatory cascades to expressing neurotoxic factors and decreasing phagocytic activity. A positive consequence in the context of MS is the release of TGF-α by microglia that can act on the astrocytic ErbB1 receptor, which was shown to limit disease progression in the EAE mouse model by releasing neuroprotective factors. Additionally, a type of microglia with an anti-inflammatory genetic signature was shown to secrete IL-10, which through its astrocytic receptor induces TGF-β secretion, inhibiting microglia activation [68, 69]. Employing the widely used murine model of MS, the cuprizone model, where oligodendrocyte death and demyelination is induced by the copper chelator cuprizone, it was observed that the recruitment of microglia by astrocytes to demyelinating lesions can be beneficial and restorative due to the phagocytic function of microglia, which removes myelin debris and enables the proliferation of oligodendrocyte precursors [70].

Oligodendrocytes respond very differently depending on the microglial population stimulating them. Microglia play a crucial role in the development of mature oligodendrocytes by secreting essential factors that foster proper homeostasis of oligodendrocyte precursors and mature cells [71]. Both activated and stationary microglia secrete factors as IGF-2 and galectin-2/3, which are responsible for oligodendrocyte precursors proliferation [72]. Thus, depending on the amount and type of factors secreted by microglia, the result is the acceleration or slowdown of oligodendrocyte precursor development. Another essential role in the context of MS is myelination, which can be influenced by the imbalance of microglial pruning of oligodendrocyte precursors, mediated by fractalkine. Additionally, microglia promote remyelination by phagocytosing myelin debris, leaving the environment conducive to the process [73]. However, excessive microglial activation can further damage OLs. For instance, activated microglia present in MS proactive lesions release TNF-α, a cytokine shown to kill oligodendrocytes in culture [74].

## 5 MICROGLIAL ROLE IN MYELIN DAMAGE AND REMYELINATION IN ANIMAL MODELS OF MS

The myelin sheath is a lipid-rich membrane that surrounds neuronal axons. Its function is to speed up the electrical signal transmitted along the axons and maintain metabolic support for neurons [75, 76]. Oligodendrocytes are the cells responsible for a process called myelination, which occurs when these cells extend their processes to the axons and wrap around them discontinuously [77]. In neurodegenerative diseases such as MS and AD, the loss of the myelin sheath, known as demyelination, can occur [78]. Demyelination happens when there is a loss of myelin around neuronal axons. In MS, demyelination occurs both due to an imbalance in the inflammatory response and the dysregulation of oligodendrocytes. Changes in the myelin sheath itself can precede demyelination, as it has been observed that in normal-appearing white matter areas of patients with chronic MS, there were already changes in the lipid profile of myelin sheath proteins [79]. In animal models and patients with MS, demyelination can occur after structural changes in the myelin or after the death of oligodendrocytes [80-83]. Interestingly, hypermyelination can also precede demyelination, as demonstrated in mice [84]. A natural regenerative process that occurs after demyelination in the mammalian CNS is remyelination. With demyelination, the axon remains intact, but remyelination is necessary; otherwise, axonal degeneration will occur [85]. In demyelinating diseases such as MS, remyelination can fail and lead to the accumulation of permanent damage to the CNS [86]. The processes involving this remyelination failure may occur due to a deficiency in the activation, recruitment, and differentiation of OPCs and neural stem cells [87-89]. Additionally, microglia can act by increasing inflammatory and neurodegenerative processes that hinder remyelination [88].

Many mechanisms can be employed by microglia to cause damage to the myelin sheath, from direct actions on oligodendrocytes to indirect actions influencing other cells that contribute to demyelination. Although myelin phagocytosis initially occurs through the action of T cells that specifically target myelin, these cells are activated by antigen-presenting cells such as microglia and dendritic cells, initiating the inflammatory cascade observed in both the EAE mouse model and MS [73]. Microglia can also cause damage to myelin by releasing nitric oxide (NO) and glutamate [73]. Moreover, using the EAE model, it has been demonstrated that microglia can directly facilitate the breakdown of the BBB by activating an inflammatory process and promoting autoimmune inflammation [90, 91]. Thus, it is evident that the actions of microglia play a fundamental role in the initiation and establishment of MS pathology.

Direct and indirect contributions of microglia to demyelination have been demonstrated in animal models of MS [7]. The inflammatory response induced by microglia is a powerful tool that affects all cells in the CNS, including oligodendrocytes. Microglia can act both to harm and to favor processes involving the myelin sheath in MS and other neurodegenerative diseases such as AD. In the cuprizone model, it was observed that microglia depletion reduces the extent of demyelinating lesions and decreases oligodendrocyte loss [92]. Furthermore, treating the cuprizone mouse model with the CSF-1 kinase activity inhibitor (BLZ94) results in decreased number of microglia, improved remyelination, and diminished demyelination, along with improved motor function [93, 94]. On the other hand, treating EAE mice with another CSF-1 receptor antagonist, PLX3397, which also leads to microglia depletion, leads to increased T cell proliferation in the CNS and worsened clinical score when the drug is administered during the chronic phase, indicating that microglia may be important for suppressing the secondary progression of autoimmune encephalomyelitis [95]. However, in the cuprizone model, treatment with PLX2297 improves the extent of demyelination, oligodendrocyte loss, and reactive astrogliosis, also improving motor recovery [86, 92]. This autoimmune inflammatory process might be aided by a non-canonical NF-κB pathway. By selectively deleting the non-canonical NF-κB-inducing kinase (NIK) in microglia, demyelination is reduced and EAE progression is halted [91]. The same does not occur in the initial phase of the model, where an inflammatory response mainly mediated by T cells is initiated. Moreover, demyelination induced by microglia can occur indirectly, through signaling by a colony-stimulating factor receptor (CSF) [91]. In the cuprizone model, selective depletion of this receptor in microglia significantly reduces demyelination and oligodendrocyte loss [92]. Thus, considering the results obtained in animal models of MS, it is not possible to be certain whether microglia play a harmful or beneficial role in myelin handling.

The phagocytic capacity of microglia is essential in the context of MS lesions, as repairing the demyelinated tissue filled with dead cells and debris requires an initial “cleaning”, allowing subsequent repair of the damage. Microglial phagocytosis can remove large myelin debris and even entire dead cells [96]. After phagocytosis, microglia must effectively process the absorbed content; otherwise, the process could aggravate the neuroinflammatory lesion [97]. In MS lesions, iron deposition can occur, leading to a series of detrimental events for various cells, including microglia. Iron is normally found in oligodendrocytes, and is important for myelin formation, as iron is a cofactor for the biosynthesis of cholesterol and lipids, essential components of myelin [98, 99]. However, in pathological situations such as MS, the destruction of oligodendrocytes and myelin results in extracellular iron deposition, leading to oxidative stress and increased polarization of microglia and macrophages towards a pro-inflammatory profile. In an attempt to avoid more damage, microglia and macrophages phagocytose the deposited iron [98]. However, iron-induced toxicity can lead to the degeneration of these myeloid cells, promoting additional waves of iron deposition and tissue damage [99]. In addition, it has been demonstrated that in chronic demyelinating lesions in the white matter, there is iron in M1 microglia/macrophages and non-phagocytic cells [100]. This is an important feature of MS pathology, as iron accumulation is detected by iron-sensitive magnetic resonance imaging (MRI) even during the early stages of MS, and this accumulation correlates positively with disease progression [101-103].

Despite being implicated in myelin sheath damage and demyelinating processes, microglia can also act to promote remyelination. The remyelination process restores the conduction of electrical impulses and redirects metabolic and trophic support to the axons. This occurs when OPCs migrate to the lesion area, proliferate, and develop into new mature myelinating oligodendrocytes. This entire process is facilitated by the actions of microglia, which release regenerative factors and phagocytose myelin debris, creating an ideal environment for OPCs to enter the remyelination process [104]. In addition to the classic remyelination process, involving newly differentiated OLs, mature OLs can also extend their processes again and remyelinate a demyelinated axon [105]. This type of remyelination can occur after OLs lose their processes following injury, although it happens to a lesser extent, resulting in inefficient and poorly directed remyelination. Moreover, the exact molecular mechanisms underlying these processes are still not fully elucidated [96].

By secreting neuroprotective and neurotrophic factors, including platelet-derived growth factors (PDGFs), vascular endothelial growth factors (VEGFs), transforming growth factors (TGFs), and insulin-like growth factor 1 (IGF1), microglia can further facilitate myelin repair by stimulating the proliferation, differentiation, and recruitment of OPCs [7]. By releasing cholesterol, microglia can help OPCs in myelinogenesis and proliferation processes [106, 107]. Another form of myelin repair is the modulation of the extracellular matrix (ECM), achieved through the secretion of various matrix metalloproteinases (MMPs) and transglutaminase 2 (TG2), which can lead to the recruitment and differentiation of OPCs [104]. Interestingly, it has been proposed that the mechanism by which microglia can assist in remyelination may be related to the aryl hydrocarbon receptor (AhR), a cytoplasmic receptor and transcription factor that operates in various physiological and pathological pathways, regulating processes such as inflammation resolution, differentiation, and immune cell function, among others [108, 109]. It has been demonstrated that repairing acute demyelinating lesions triggered by cuprizone can occur through sterol release by microglia [9]. Furthermore, AhR deletion in microglia impairs remyelination and causes the accumulation of myelin debris by impairing microglial phagocytic function [110]. Another important receptor is the triggering receptor expressed on myeloid cells 2 (TREM2) expressed by microglia, whose activation leads to remyelination and increased removal of myelin debris in the CPZ model [111]. In the lysophosphatidylcholine-induced demyelination model, it was demonstrated that microglia depletion reduces the recruitment and proliferation of OPCs, resulting in impaired remyelination [112].

Microglial actions on the myelin sheath are not exclusively beneficial or harmful, as they can be ambiguous. Using a mutant mouse model with defective PLP protein, Groh *et al*. (2023) demonstrated that microglial function in demyelination is more complex than previously thought. Axonal degeneration and loss of function occurring in demyelinating diseases such as MS are primarily attributed to myelin loss. However, it has been shown that persistent coating with disturbed myelin poses a risk of axonal degeneration, neuronal loss, and behavioral decline [113]. Moreover, it appears that axonal damage caused by CD8^+^ T cells is less likely to result in progressive degeneration when activated microglia efficiently mediate demyelination [113]. Thus, the role of microglia in remyelination/demyelination is controversial and more studies are needed to elucidate the mechanisms underlying these processes, especially because myelin alterations in MS are an important target for the development of effective therapies to treat patients.

## 6 EVALUATION OF MICROGLIA IN CLINICAL PRACTICE

Given the importance of microglial activation in the pathophysiology of MS and the correlation between activated microglia patterns and the phases of MS, evaluating microglia is pointed as a potential strategy to facilitate the clinical assessment of a patient's disease stage.

Susceptibility-weighted MRI sequences enable the visualization of iron deposition sites, distinguishing iron concentrations and differentiating iron from calcium [110]. Myelin loss and consequent loss of lipid macromolecules increase the region of magnetic susceptibility values, allowing iron rim lesion detection in MS using MRI [101]. These lesions correlate with iron and CD68^+^ microglia/macrophages distribution in histopathological analysis, serving as a biomarker for chronic lesion activity in MS (Fig. **2A**) [101, 114]. Additionally, the C1q, a crucial part of the classical complement pathway involved in the immune response, is expressed by microglia at the edges of chronic active lesions. This expression has been identified as a significant factor in driving inflammation. The presence of C1q in these regions further supports its potential as a biomarker for monitoring the progression of chronic lesions in MS [115].

An additional method for assessing microglial activation involves positron emission tomography (PET) with radiotracer targeting the translocator protein (TSPO), a protein located on the mitochondrial membrane that is upregulated during microglial activation [116]. The tracer 11C-(R)-PK11195 exhibits high affinity to TSPO in MS active/inflammatory lesions, which reduces post-treatment with highly effective drugs [117, 118]. Of note, significant binding in normal-appearing white matter is more common in SPMS than in the RRMS [119].

Another biomarker studied in MS is neurofilament light (NfL), a protein found in neurons that is a component of neurofilaments, essential for the maintenance of axonal structure and function. Elevations in NfL levels are observed in various neurological diseases, including MS. Recent studies indicate that NfL levels may be useful for assessing disease activity and treatment response in patients with MS [120]. There appears to be an association between serum NfL levels (sNfL) and microglial activation measurable by PET in the brains of MS patients, such that the higher the distribution volume of 11C-(R)-PK11195, the higher the level of sNfL. This fact demonstrates the importance of active chronic lesions in promoting neuroaxonal damage [121].

## 7 DISEASE-MODIFYING THERAPIES AND MICROGLIA

Over the past two decades, more than a dozen drugs were approved for MS treatment, particularly for the relapsing forms [122]. In general, these drugs act by either depleting immune cells in the periphery or preventing their transmigration into the CNS. It has been shown that disease-modifying therapies (DMTs) can potentially affect microglia directly or indirectly. However, the extent and significance of these effects on microglia remain unclear and require further investigation [123]. Medications that do not cross the BBB, such as IFN-β and glatiramer acetate, act on microglia by causing a change in the pattern of peripheral T lymphocytes. Meanwhile, oral medications like fingolimod access the CNS and act directly on microglia. The advent of immunobiological therapies has revolutionized the management of MS, offering more targeted approaches to modulate the immune system and reduce disease activity. These medications work by specifically targeting components of the immune system, thereby decreasing the frequency and severity of relapses and slowing the progression of disability [124]. However, the level of monoclonal antibodies found in the CNS is low, so their direct action on microglia is questionable. Probably, similar to IFN-β and glatiramer acetate, medications such as natalizumab, ocrelizumab, ofatumumab, and alemtuzumab will show only indirect effects on microglia.

IFN-β is a subcutaneously or intramuscularly administered DMT approved for the treatment of RRMS, but without positive results for the treatment of progressive phases, as shown in the case of SPMS [125, 126]. However, IFN-β acts on peripheral T cells by promoting a shift towards the Th2 profile, in addition to promoting the production of B-cell activation factor (BAFF). Thus, the lymphocytes reaching the CNS exhibit a profile with a lower predisposition to activate a neurotoxic type of microglia [127]. The same shift in T lymphocyte profile to Th2 occurs with the use of glatiramer acetate, an immunomodulatory medication composed of a polymer of four amino acids (glutamic acid, lysine, alanine, tyrosine). Additionally, glatiramer acetate may promote neuroprotection by inducing the secretion of neurotrophic factors from T cells, further contributing to its therapeutic effects in MS [123, 128]. Another medication that also allows for greater activation of the Th2 pattern with the production of IL-4 and IL-5 is the oral medication dimethyl fumarate. However, this drug is capable of crossing the BBB and within the CNS it induces an antioxidant response and an inhibitory effect on pro-inflammatory molecules [129, 130]. Therefore, all these drugs might affect microglia indirectly.

Teriflunomide is the active metabolite of leflunomide, which acts by altering pyrimidine synthesis [131]. This is a crucial point for the metabolism of cells with a high proliferation rate, including active T and B lymphocytes [132]. As mentioned, some oral medications can cross the BBB and possibly exert a direct action on microglia. However, there is no evidence that teriflunomide crosses the BBB [132]. Thus, this drug could act on microglia indirectly. Meanwhile, fingolimod, a sphingosine-1-phosphate (S1P) receptor modulator, is highly lipophilic and can cross the BBB, reaching the cerebral white matter [133]. In a phase 3 clinical study for RRMS, it was observed that fingolimod reduced disease progression and brain atrophy [134]. However, it failed to demonstrate similar outcomes for patients with PPMS [135]. The main mechanism of action of fingolimod occurs peripherally by preventing the flow of lymphocytes out of the lymph node, dependent on the S1P gradient [136]. However, S1P receptors are present in CNS cells in different distribution patterns. Fingolimod binds to all S1P receptor subtypes, except subtype 4 (S1P4R). It is well known that microglia mainly express S1PR1, S1PR3, and S1PR5 receptors [137]. Evaluation in mice suggests that fingolimod binding to S1PR1 down-regulates the production of pro-inflammatory cytokines, such as IL-1, IL-6, and TNF-α in activated microglia. Additionally, it up-regulates the production of BDNF, thus modulating microglial activation for the production of remyelination markers [138]. Siponimod, a medication approved for the treatment of RRMS and SPMS, is also an S1P modulator, but selective for S1PR1 and S1PR5 receptors [139]. The main mechanism of action of this drug is also related to lymphocyte flow through the lymph node through the S1P gradient. However, Gruchot *et al*., using murine-derived microglia culture, demonstrated that siponimod prevents microglia from altering their cytoskeleton to activated forms, reduces the expression of induced Nitric Oxide Synthase (iNOS) protein, and modulates cytokine production by microglia, preventing TNF-α upregulation [140]. TNF-α increases glutamatergic excitotoxicity, enhances neuroinflammatory response, and can induce oligodendrocyte death [141]. In MS, TNF-α levels are directly correlated with increased disability and a higher risk of disease progression [142]. Thus, siponimod positive effects on MS may also rely on its direct actions on microglia.

Natalizumab was the first monoclonal antibody approved for the treatment of MS [143]. Its mechanism of action involves reducing the passage of lymphocytes across the BBB by binding to the α-4-β-1 integrin receptor, which interacts with cell surface adhesion molecules (CAM) and facilitates the initiation of the cell diapedesis process [144]. Although not found in the CNS, natalizumab leads to a reduction in the binding of the radiotracer 11C-(R)-PK11195 to TSPO in patients [118, 145]. Ocrelizumab, ofatumumab, and ublituximab are monoclonal antibodies approved for MS treatment that act on B lymphocyte CD20 receptors, resulting in the depletion of these cells [146-150]. Rituximab also has a similar mechanism of action, with efficacy already described in the literature for controlling MS activity, although it is not approved by the Food and Drug Administration (FDA) and European Medicines Agency (EMA) [151]. Throughout the pathophysiological evolution of MS, tertiary lymphoid tissue follicles containing B lymphocytes develop, leading to “compartmentalized inflammation” in the CNS [152]. These tertiary follicles are located in the subpial region, but also in some perivascular regions. Despite positive results demonstrating a reduction in disability progression following treatment with these medications, it is not certain that they can act on the lymphoid tissues and interrupt the inflammatory process [153]. Another monoclonal antibody approved by the FDA for MS treatment is alemtuzumab, which targets CD52 expressed on the cell surface of myeloid cells, B lymphocytes, and T lymphocytes [154]. During lymphocyte reconstitution in RRMS patients treated with alemtuzumab, it is observed an increase in the production of neuroprotective factors, such as BDNF [155]. In summary, while the primary mechanisms of immunobiological treatments involve modulating lymphocyte activity and reducing inflammation in MS, these therapies may also indirectly influence microglial activity by altering the overall inflammatory milieu within the CNS. This indirect modulation could potentially affect microglial activation states and their role in neuroinflammation, thereby contributing to the therapeutic effects observed in MS treatment.

Cladribine is a synthetic analog of deoxyadenosine, given to patients orally, that selectively depletes lymphocytes. Cladribine is capable of crossing the BBB and acting directly on resident microglia [156]. A study with primary cultured murine microglia demonstrated that only activated microglia are affected by cladribine actions. Additionally, treatment with this drug leads to an increase in the production of anti-inflammatory mediators, suggesting that microglia acquire a less active phenotype when treated with cladribine [157].

The Bruton tyrosine kinase (BTK) is an important intracellular signaling molecule involved in the maturation, proliferation, survival, and activation of B and myeloid cells [158]. Preclinical studies have shown that BTK inhibition can reduce microglial activation and, therefore, there was significant anticipation regarding the potential of this molecule for MS treatment [159]. However, the EVOLUTION study evaluating the efficacy of evobrutinib (a BTK inhibitor), as compared to teriflunomide, was discontinued due to not achieving the primary outcome of reducing the annualized relapse rate [160].

In summary, while the primary mechanisms of immunobiological treatments involve modulating lymphocyte activity and reducing inflammation in MS, these therapies may also produce positive effects on MS by indirectly influencing microglial activity by altering the overall inflammatory milieu within the CNS. Ongoing research on the impact of these medications on microglia is essential to fully understand their therapeutic potential, improve existing treatment strategies, and develop new approaches to control inflammation and neurodegeneration in MS.

## CONCLUSION

Microglia have a central function in MS pathology, being active at all disease stages. These cells play multifaceted roles in MS, as their initial response is protective, most likely trying to limit insult to the CNS. However, over time, exacerbated activation of microglia may also lead to elevated expression of harmful molecules that drive neuroinflammation and contribute to MS neuropathology. On one hand, microglia can secrete factors that play a crucial role in the development of oligodendrocytes and reduce neuroinflammation by phagocytosing myelin debris, thus aiding in proper remyelination. On the other hand, in MS lesions, these cells release cytokines that can activate astrocytes and immune cells, triggering the expression of neurotoxic factors and exacerbating neuroinflammation. Although it is difficult to be certain whether these cells are friends or foes, there is no doubt that microglia will be a relevant target for MS diagnosis and treatment in the future. MRI and PET scans allow evaluation of microglia/macrophage biomarkers, facilitating the clinical assessment of a patient's disease stage. Moreover, new microglia-specific markers are being discovered, which will increase diagnostic precision, helping to identify active and chronic MS lesions. Because microglia are present in all MS phases, these cells are also an important drug target. However, it is important to mention that the development of effective therapies targeting microglia depends on a well-refined characterization of microglia activation patterns, as we now know that there are several populations of microglia, whose origination depends on their surroundings, activating molecules, and disease context.

## LIST OF ABBREVIATIONS

AD: Alzheimer's Disease
ALS: Amyotrophic Lateral Sclerosis
BBB: Blood-brain Barrier
BDNF: Brain-derived Neurotrophic Factor
BTK: Bruton Tyrosine Kinase
CNS: Central Nervous System
CIS: Clinically Isolated Syndrome
CSF-1: Colony-stimulating Factor 1
DMTs: Disease-modifying Therapies
DMTs: Disease-modifying Treatments
ECM: Extracellular Matrix
HLA: Human Leukocyte Antigen
iNOS: Induced Nitric Oxide Synthase
IGF1: Insulin-like Growth Factor 1
IL-17: Interleukin-17
MRI: Magnetic Resonance Imaging
MMPs: Matrix Metalloproteinases
MS: Multiple Sclerosis
MBP: Myelin Basic Protein
MAG: Myelin-associated Glycoprotein
NBB: Netherlands Brain Bank
NO: Nitric Oxide
PDGFs: Platelet-derived Growth Factors
PET: Positron Emission Tomography
PLP: Proteolipid Protein
SDT: Symbol-digit Test
TLRs: Toll-like Receptors
TGFs: Transforming Growth Factors
TNF-α: Tumor Necrosis Factor-alpha
VEGFs: Vascular Endothelial Growth Factors

## References

[1] Mosser C.A., Baptista S., Arnoux I., Audinat E. (2017). Microglia in CNS development: Shaping the brain for the future.. Prog. Neurobiol..

[2] Ginhoux F., Greter M., Leboeuf M., Nandi S., See P., Gokhan S., Mehler M.F., Conway S.J., Ng L.G., Stanley E.R., Samokhvalov I.M., Merad M. (2010). Fate mapping analysis reveals that adult microglia derive from primitive macrophages.. Science.

[3] Bruttger J., Karram K., Wörtge S., Regen T., Marini F., Hoppmann N., Klein M., Blank T., Yona S., Wolf Y., Mack M., Pinteaux E., Müller W., Zipp F., Binder H., Bopp T., Prinz M., Jung S., Waisman A. (2015). Genetic cell ablation reveals clusters of local self-renewing microglia in the mammalian central nervous system.. Immunity.

[4] Erblich B., Zhu L., Etgen A.M., Dobrenis K., Pollard J.W. (2011). Absence of colony stimulation factor-1 receptor results in loss of microglia, disrupted brain development and olfactory deficits.. PLoS One.

[5] Spittau B., Dokalis N., Prinz M. (2020). The role of TGFβ signaling in microglia maturation and activation.. Trends Immunol..

[6] Gosselin D., Skola D., Coufal N.G., Holtman I.R., Schlachetzki J.C.M., Sajti E., Jaeger B.N., O’Connor C., Fitzpatrick C., Pasillas M.P., Pena M., Adair A., Gonda D.D., Levy M.L., Ransohoff R.M., Gage F.H., Glass C.K. (2017). An environment-dependent transcriptional network specifies human microglia identity.. Science.

[7] Distéfano-Gagné F., Bitarafan S., Lacroix S., Gosselin D. (2023). Roles and regulation of microglia activity in multiple sclerosis: Insights from animal models.. Nat. Rev. Neurosci..

[8] Lawson L.J., Perry V.H., Dri P., Gordon S. (1990). Heterogeneity in the distribution and morphology of microglia in the normal adult mouse brain.. Neuroscience.

[9] Askew K., Li K., Olmos-Alonso A., Garcia-Moreno F., Liang Y., Richardson P., Tipton T., Chapman M.A., Riecken K., Beccari S., Sierra A., Molnár Z., Cragg M.S., Garaschuk O., Perry V.H., Gomez-Nicola D. (2017). Coupled proliferation and apoptosis maintain the rapid turnover of microglia in the adult brain.. Cell Rep..

[10] Plemel J.R., Stratton J.A., Michaels N.J., Rawji K.S., Zhang E., Sinha S., Baaklini C.S., Dong Y., Ho M., Thorburn K., Friedman T.N., Jawad S., Silva C., Caprariello A.V., Hoghooghi V., Yue J., Jaffer A., Lee K., Kerr B.J., Midha R., Stys P.K., Biernaskie J., Yong V.W. (2020). Microglia response following acute demyelination is heterogeneous and limits infiltrating macrophage dispersion.. Sci. Adv..

[11] Djalali S., Höltje M., Große G., Rothe T., Stroh T., Große J., Deng D.R., Hellweg R., Grantyn R., Hörtnagl H., Ahnert-Hilger G. (2005). Effects of brain‐derived neurotrophic factor (BDNF) on glial cells and serotonergic neurones during development.. J. Neurochem..

[12] Wlodarczyk A., Holtman I.R., Krueger M., Yogev N., Bruttger J., Khorooshi R., Benmamar-Badel A., de Boer-Bergsma J.J., Martin N.A., Karram K., Kramer I., Boddeke E.W.G.M., Waisman A., Eggen B.J.L., Owens T. (2017). A novel microglial subset plays a key role in myelinogenesis in developing brain.. EMBO J..

[13] Parkhurst C.N., Yang G., Ninan I., Savas J.N., Yates J.R., Lafaille J.J., Hempstead B.L., Littman D.R., Gan W.B. (2013). Microglia promote learning-dependent synapse formation through brain-derived neurotrophic factor.. Cell.

[14] Prinz M., Masuda T., Wheeler M.A., Quintana F.J. (2021). Microglia and central nervous system–associated macrophages—from origin to disease modulation.. Annu. Rev. Immunol..

[15] Hickman S.E., Kingery N.D., Ohsumi T.K., Borowsky M.L., Wang L., Means T.K., El Khoury J. (2013). The microglial sensome revealed by direct RNA sequencing.. Nat. Neurosci..

[16] Färber K., Kettenmann H. (2006). Functional role of calcium signals for microglial function.. Glia.

[17] El Khoury J.B., Moore K.J., Means T.K., Leung J., Terada K., Toft M., Freeman M.W., Luster A.D. (2003). CD36 mediates the innate host response to beta-amyloid.. J. Exp. Med..

[18] Hickman S., Izzy S., Sen P., Morsett L., El Khoury J. (2018). Microglia in neurodegeneration.. Nat. Neurosci..

[19] Umpierre A.D., Wu L.J. (2021). How microglia sense and regulate neuronal activity.. Glia.

[20] Wendimu M.Y., Hooks S.B. (2022). Microglia phenotypes in aging and neurodegenerative diseases.. Cells.

[21] Gao C., Jiang J., Tan Y., Chen S. (2023). Microglia in neurodegenerative diseases: Mechanism and potential therapeutic targets.. Signal Transduct. Target. Ther..

[22] Tang Y., Le W. (2016). Differential roles of M1 and M2 microglia in neurodegenerative diseases.. Mol. Neurobiol..

[23] Liddelow S.A., Guttenplan K.A., Clarke L.E., Bennett F.C., Bohlen C.J., Schirmer L., Bennett M.L., Münch A.E., Chung W.S., Peterson T.C., Wilton D.K., Frouin A., Napier B.A., Panicker N., Kumar M., Buckwalter M.S., Rowitch D.H., Dawson V.L., Dawson T.M., Stevens B., Barres B.A. (2017). Neurotoxic reactive astrocytes are induced by activated microglia.. Nature.

[24] Ransohoff R.M. (2016). A polarizing question: do M1 and M2 microglia exist?. Nat. Neurosci..

[25] Lassmann H., van Horssen J., Mahad D. (2012). Progressive multiple sclerosis: pathology and pathogenesis.. Nat. Rev. Neurol..

[26] van der Poel M., Ulas T., Mizee M.R., Hsiao C.C., Miedema S.S.M., Adelia S.K.G., Helder B., Tas S.W., Schultze J.L., Hamann J., Huitinga I. (2019). Transcriptional profiling of human microglia reveals grey-white matter heterogeneity and multiple sclerosis-associated changes.. Nat. Commun..

[27] Nimmerjahn A., Kirchhoff F., Helmchen F. (2005). Resting microglial cells are highly dynamic surveillants of brain parenchyma in vivo.. Science.

[28] Hellwig S., Brioschi S., Dieni S., Frings L., Masuch A., Blank T., Biber K. (2016). Altered microglia morphology and higher resilience to stress-induced depression-like behavior in CX3CR1-deficient mice.. Brain Behav. Immun..

[29] Hui C.W., St-Pierre M.K., Detuncq J., Aumailley L., Dubois M.J., Couture V., Skuk D., Marette A., Tremblay J.P., Lebel M., Tremblay M.È. (2018). Nonfunctional mutant Wrn protein leads to neurological deficits, neuronal stress, microglial alteration, and immune imbalance in a mouse model of Werner syndrome.. Brain Behav. Immun..

[30] Keren-Shaul H., Spinrad A., Weiner A., Matcovitch-Natan O., Dvir-Szternfeld R., Ulland T.K., David E., Baruch K., Lara-Astaiso D., Toth B., Itzkovitz S., Colonna M., Schwartz M., Amit I. (2017). A Unique microglia type associated with restricting development of Alzheimer’s disease.. Cell.

[31] Lee D.C., Rizer J., Selenica M.L.B., Reid P., Kraft C., Johnson A., Blair L., Gordon M.N., Dickey C.A., Morgan D. (2010). LPS- induced inflammation exacerbates phospho-tau pathology in rTg4510 mice.. J. Neuroinflammation.

[32] Andreadou M., Ingelfinger F., De Feo D., Cramer T.L.M., Tuzlak S., Friebel E., Schreiner B., Eede P., Schneeberger S., Geesdorf M., Ridder F., Welsh C.A., Power L., Kirschenbaum D., Tyagarajan S.K., Greter M., Heppner F.L., Mundt S., Becher B. (2023). IL-12 sensing in neurons induces neuroprotective CNS tissue adaptation and attenuates neuroinflammation in mice.. Nat. Neurosci..

[33] Cossburn M., Ingram G., Hirst C., Ben-Shlomo Y., Pickersgill T.P., Robertson N.P. (2012). Age at onset as a determinant of presenting phenotype and initial relapse recovery in multiple sclerosis.. Mult. Scler..

[34] Olsson T., Barcellos L.F., Alfredsson L. (2017). Interactions between genetic, lifestyle and environmental risk factors for multiple sclerosis.. Nat. Rev. Neurol..

[35] Attfield K.E., Jensen L.T., Kaufmann M., Friese M.A., Fugger L. (2022). The immunology of multiple sclerosis.. Nat. Rev. Immunol..

[36] Croxford A.L., Spath S., Becher B. (2015). GM-CSF in neuroinflammation: Licensing myeloid cells for tissue damage.. Trends Immunol..

[37] Serafini B., Rosicarelli B., Magliozzi R., Stigliano E., Aloisi F. (2004). Detection of ectopic B-cell follicles with germinal centers in the meninges of patients with secondary progressive multiple sclerosis.. Brain Pathol..

[38] Wekerle H. (2017). B cells in multiple sclerosis.. Autoimmunity.

[39] Yong V.W. (2022). Microglia in multiple sclerosis: Protectors turn destroyers.. Neuron.

[40] Fox E.J. (2004). Immunopathology of multiple sclerosis.. Neurology.

[41] Lublin F.D., Reingold S.C., Cohen J.A., Cutter G.R., Sørensen P.S., Thompson A.J., Wolinsky J.S., Balcer L.J., Banwell B., Barkhof F., Bebo B., Calabresi P.A., Clanet M., Comi G., Fox R.J., Freedman M.S., Goodman A.D., Inglese M., Kappos L., Kieseier B.C., Lincoln J.A., Lubetzki C., Miller A.E., Montalban X., O’Connor P.W., Petkau J., Pozzilli C., Rudick R.A., Sormani M.P., Stüve O., Waubant E., Polman C.H. (2014). Defining the clinical course of multiple sclerosis.. Neurology.

[42] Kalincik T., Buzzard K., Jokubaitis V., Trojano M., Duquette P., Izquierdo G., Girard M., Lugaresi A., Grammond P., Grand’Maison F., Oreja-Guevara C., Boz C., Hupperts R., Petersen T., Giuliani G., Iuliano G., Lechner-Scott J., Barnett M., Bergamaschi R., Van Pesch V., Amato M.P., van Munster E., Fernandez-Bolanos R., Verheul F., Fiol M., Cristiano E., Slee M., Rio M.E., Spitaleri D., Alroughani R., Gray O., Saladino M.L., Flechter S., Herbert J., Cabrera-Gomez J.A., Vella N., Paine M., Shaw C., Moore F., Vucic S., Savino A., Singhal B., Petkovska-Boskova T., Parratt J., Sirbu C.A., Rozsa C., Liew D., Butzkueven H. (2014). Risk of relapse phenotype recurrence in multiple sclerosis.. Mult. Scler..

[43] Thompson A.J., Banwell B.L., Barkhof F., Carroll W.M., Coetzee T., Comi G., Correale J., Fazekas F., Filippi M., Freedman M.S., Fujihara K., Galetta S.L., Hartung H.P., Kappos L., Lublin F.D., Marrie R.A., Miller A.E., Miller D.H., Montalban X., Mowry E.M., Sorensen P.S., Tintoré M., Traboulsee A.L., Trojano M., Uitdehaag B.M.J., Vukusic S., Waubant E., Weinshenker B.G., Reingold S.C., Cohen J.A. (2018). Diagnosis of multiple sclerosis: 2017 revisions of the McDonald criteria.. Lancet Neurol..

[44] Müller J., Cagol A., Lorscheider J., Tsagkas C., Benkert P., Yaldizli Ö., Kuhle J., Derfuss T., Sormani M.P., Thompson A., Granziera C., Kappos L. (2023). Harmonizing definitions for progression independent of relapse activity in multiple sclerosis.. JAMA Neurol..

[45] Galea I., Ward-Abel N., Heesen C. (2015). Relapse in multiple sclerosis.. BMJ.

[46] Lublin F.D., Häring D.A., Ganjgahi H., Ocampo A., Hatami F., Čuklina J., Aarden P., Dahlke F., Arnold D.L., Wiendl H., Chitnis T., Nichols T.E., Kieseier B.C., Bermel R.A. (2022). How patients with multiple sclerosis acquire disability.. Brain.

[47] Guerrero B.L., Sicotte N.L. (2020). Microglia in multiple sclerosis: Friend or foe?. Front. Immunol..

[48] Kuhlmann T., Ludwin S., Prat A., Antel J., Brück W., Lassmann H. (2017). An updated histological classification system for multiple sclerosis lesions.. Acta Neuropathol..

[49] Lucchinetti C., Brück W., Parisi J., Scheithauer B., Rodriguez M., Lassmann H. (2000). Heterogeneity of multiple sclerosis lesions: Implications for the pathogenesis of demyelination.. Ann. Neurol..

[50] Frischer J.M., Weigand S.D., Guo Y., Kale N., Parisi J.E., Pirko I., Mandrekar J., Bramow S., Metz I., Brück W., Lassmann H., Lucchinetti C.F. (2015). Clinical and pathological insights into the dynamic nature of the white matter multiple sclerosis plaque.. Ann. Neurol..

[51] Luchetti S., Fransen N.L., van Eden C.G., Ramaglia V., Mason M., Huitinga I. (2018). Progressive multiple sclerosis patients show substantial lesion activity that correlates with clinical disease severity and sex: a retrospective autopsy cohort analysis.. Acta Neuropathol..

[52] Zhang X., Chen F., Sun M., Wu N., Liu B., Yi X., Ge R., Fan X. (2023). Microglia in the context of multiple sclerosis.. Front. Neurol..

[53] Matejuk A., Ransohoff R.M. (2020). Crosstalk between astrocytes and microglia: An overview.. Front. Immunol..

[54] Marinelli S., Basilico B., Marrone M.C., Ragozzino D. (2019). Microglia-neuron crosstalk: Signaling mechanism and control of synaptic transmission.. Semin. Cell Dev. Biol..

[55] Allen N.J., Lyons D.A. (2018). Glia as architects of central nervous system formation and function.. Science.

[56] Frost J.L., Schafer D.P. (2016). Microglia: Architects of the developing nervous system.. Trends Cell Biol..

[57] Liu Y.J., Spangenberg E.E., Tang B., Holmes T.C., Green K.N., Xu X. (2021). Microglia elimination increases neural circuit connectivity and activity in adult mouse cortex.. J. Neurosci..

[58] Ronzano R., Roux T., Thetiot M., Aigrot M.S., Richard L., Lejeune F.X., Mazuir E., Vallat J.M., Lubetzki C., Desmazières A. (2021). Microglia-neuron interaction at nodes of Ranvier depends on neuronal activity through potassium release and contributes to remyelination.. Nat. Commun..

[59] Zhou M., Cornell J., Salinas S., Huang H-Y. (2022). Microglia regulation of synaptic plasticity and learning and memory.. Neural Regen. Res..

[60] Lyons A., Downer E.J., Crotty S., Nolan Y.M., Mills K.H.G., Lynch M.A. (2007). CD200 ligand receptor interaction modulates microglial activation *in vivo* and in vitro: A role for IL-4.. J. Neurosci..

[61] Dissing-Olesen L., LeDue J.M., Rungta R.L., Hefendehl J.K., Choi H.B., MacVicar B.A. (2014). Activation of neuronal NMDA receptors triggers transient ATP-mediated microglial process outgrowth.. J. Neurosci..

[62] Centonze D., Muzio L., Rossi S., Cavasinni F., De Chiara V., Bergami A., Musella A., D’Amelio M., Cavallucci V., Martorana A., Bergamaschi A., Cencioni M.T., Diamantini A., Butti E., Comi G., Bernardi G., Cecconi F., Battistini L., Furlan R., Martino G. (2009). Inflammation triggers synaptic alteration and degeneration in experimental autoimmune encephalomyelitis.. J. Neurosci..

[63] Di Filippo M., de Iure A., Giampà C., Chiasserini D., Tozzi A., Orvietani P.L., Ghiglieri V., Tantucci M., Durante V., Quiroga-Varela A., Mancini A., Costa C., Sarchielli P., Fusco F.R., Calabresi P. (2016). Persistent activation of microglia and NADPH oxidase drive hippocampal dysfunction in experimental multiple sclerosis.. Sci. Rep..

[64] Musella A., Gentile A., Guadalupi L., Rizzo F.R., De Vito F., Fresegna D., Bruno A., Dolcetti E., Vanni V., Vitiello L., Bullitta S., Sanna K., Caioli S., Balletta S., Nencini M., Buttari F., Stampanoni B.M., Centonze D., Mandolesi G. (2020). Central modulation of selective sphingosine-1-phosphate receptor 1 ameliorates experimental multiple sclerosis.. Cells.

[65] Gentile A., Musella A., De Vito F., Fresegna D., Bullitta S., Rizzo F.R., Centonze D., Mandolesi G. (2018). Laquinimod ameliorates excitotoxic damage by regulating glutamate re-uptake.. J. Neuroinflammation.

[66] Rossi S., Lo Giudice T., De Chiara V., Musella A., Studer V., Motta C., Bernardi G., Martino G., Furlan R., Martorana A., Centonze D. (2012). Oral fingolimod rescues the functional deficits of synapses in experimental autoimmune encephalomyelitis.. Br. J. Pharmacol..

[67] Parodi B., Rossi S., Morando S., Cordano C., Bragoni A., Motta C., Usai C., Wipke B.T., Scannevin R.H., Mancardi G.L., Centonze D., Kerlero de Rosbo N., Uccelli A. (2015). Fumarates modulate microglia activation through a novel HCAR2 signaling pathway and rescue synaptic dysregulation in inflamed CNS.. Acta Neuropathol..

[68] Linnerbauer M., Wheeler M.A., Quintana F.J. (2020). Astrocyte crosstalk in CNS inflammation.. Neuron.

[69] Norden D.M., Fenn A.M., Dugan A., Godbout J.P. (2014). TGFβ produced by IL‐10 redirected astrocytes attenuates microglial activation.. Glia.

[70] Sen M.K., Mahns D.A., Coorssen J.R., Shortland P.J. (2022). The roles of microglia and astrocytes in phagocytosis and myelination: Insights from the cuprizone model of multiple sclerosis.. Glia.

[71] Hagemeyer N., Hanft K.M., Akriditou M.A., Unger N., Park E.S., Stanley E.R., Staszewski O., Dimou L., Prinz M. (2017). Microglia contribute to normal myelinogenesis and to oligodendrocyte progenitor maintenance during adulthood.. Acta Neuropathol..

[72] Psenicka M.W., Smith B.C., Tinkey R.A., Williams J.L. (2021). Connecting neuroinflammation and neurodegeneration in multiple sclerosis: are oligodendrocyte precursor cells a nexus of disease?. Front. Cell. Neurosci..

[73] Kalafatakis I., Karagogeos D. (2021). Oligodendrocytes and microglia: Key players in myelin development, damage and repair.. Biomolecules.

[74] van Horssen J., Singh S., van der Pol S., Kipp M., Lim J.L., Peferoen L., Gerritsen W., Kooi E.J., Witte M.E., Geurts J.J.G., de Vries H.E., Peferoen-Baert R., van den Elsen P.J., van der Valk P., Amor S. (2012). Clusters of activated microglia in normal-appearing white matter show signs of innate immune activation.. J. Neuroinflammation.

[75] Fünfschilling U., Supplie L.M., Mahad D., Boretius S., Saab A.S., Edgar J., Brinkmann B.G., Kassmann C.M., Tzvetanova I.D., Möbius W., Diaz F., Meijer D., Suter U., Hamprecht B., Sereda M.W., Moraes C.T., Frahm J., Goebbels S., Nave K.A. (2012). Glycolytic oligodendrocytes maintain myelin and long-term axonal integrity.. Nature.

[76] Saab A.S., Tzvetavona I.D., Trevisiol A., Baltan S., Dibaj P., Kusch K., Möbius W., Goetze B., Jahn H.M., Huang W., Steffens H., Schomburg E.D., Pérez-Samartín A., Pérez-Cerdá F., Bakhtiari D., Matute C., Löwel S., Griesinger C., Hirrlinger J., Kirchhoff F., Nave K.A. (2016). Oligodendroglial NMDA receptors regulate glucose import and axonal energy metabolism.. Neuron.

[77] Simons M., Nave K.A. (2016). Oligodendrocytes: Myelination and axonal support.. Cold Spring Harb. Perspect. Biol..

[78] Boukhvalova M.S., Kastrukoff L., Blanco J.C.G. (2023). Alzheimer’s disease and multiple sclerosis: A possible connection through the viral demyelinating neurodegenerative trigger (vDENT).. Front. Aging Neurosci..

[79] Poon K.W.C., Brideau C., Klaver R., Schenk G.J., Geurts J.J., Stys P.K. (2018). Lipid biochemical changes detected in normal appearing white matter of chronic multiple sclerosis by spectral coherent Raman imaging.. Chem. Sci. (Camb.).

[80] Traka M., Podojil J.R., McCarthy D.P., Miller S.D., Popko B. (2016). Oligodendrocyte death results in immune-mediated CNS demyelination.. Nat. Neurosci..

[81] Erik S., Mar Bosch-Queralt, Julia M.E., Maria L., Judith S., Niko F., Theresa K., Peter W., Stefan A.B., Tilo R., Martin K., Markus M., Wiebke M., Alonso Barrantes-Freer, Jens S., Ting S., Gesine S., Markus H.S., Christoph W., Maximilian F., Marco P., Daniel S.R., Alexander F., Christine S., Robert F., Klaus-Armin N., Ruth M.S. (2023). Myelin insulation as a risk factor for axonal degeneration in autoimmune demyelinating disease.. Nat Neurosci.

[82] Chapman T.W., Olveda G.E., Bame X., Pereira E., Hill R.A. (2023). Oligodendrocyte death initiates synchronous remyelination to restore cortical myelin patterns in mice.. Nat. Neurosci..

[83] Romanelli E., Merkler D., Mezydlo A., Weil M.T., Weber M.S., Nikić I., Potz S., Meinl E., Matznick F.E.H., Kreutzfeldt M., Ghanem A., Conzelmann K.K., Metz I., Brück W., Routh M., Simons M., Bishop D., Misgeld T., Kerschensteiner M. (2016). Myelinosome formation represents an early stage of oligodendrocyte damage in multiple sclerosis and its animal model.. Nat. Commun..

[84] Aber E.R., Griffey C.J., Davies T., Li A.M., Yang Y.J., Croce K.R., Goldman J.E., Grutzendler J., Canman J.C., Yamamoto A. (2022). Oligodendroglial macroautophagy is essential for myelin sheath turnover to prevent neurodegeneration and death.. Cell Rep..

[85] Franklin R.J.M. (2017). ffrench-Constant, C. Regenerating CNS myelin - from mechanisms to experimental medicines.. Nat. Rev. Neurosci..

[86] Lubetzki C., Zalc B., Williams A., Stadelmann C., Stankoff B. (2020). Remyelination in multiple sclerosis: From basic science to clinical translation.. Lancet Neurol..

[87] Kremer D., Aktas O., Hartung H.P., Küry P. (2011). The complex world of oligodendroglial differentiation inhibitors.. Ann. Neurol..

[88] Gruchot J., Weyers V., Göttle P., Förster M., Hartung H.P., Küry P., Kremer D. (2019). The molecular basis for remyelination failure in multiple sclerosis.. Cells.

[89] Kuhlmann T., Miron V., Cuo Q., Wegner C., Antel J., Brück W. (2008). Differentiation block of oligodendroglial progenitor cells as a cause for remyelination failure in chronic multiple sclerosis.. Brain.

[90] Yu Z., Fang X., Liu W., Sun R., Zhou J., Pu Y., Zhao M., Sun D., Xiang Z., Liu P., Ding Y., Cao L., He C. (2022). Microglia regulate blood-brain barrier integrity *via* MiR‐126a‐5p/MMP9 axis during inflammatory demyelination.. Adv. Sci. (Weinh.).

[91] Jie Z., Ko C.J., Wang H., Xie X., Li Y., Gu M., Zhu L., Yang J.Y., Gao T., Ru W., Tang S.J., Cheng X., Sun S.C. (2021). Microglia promote autoimmune inflammation *via* the noncanonical NF-κB pathway.. Sci. Adv..

[92] Marzan D.E., Brügger-Verdon V., West B.L., Liddelow S., Samanta J., Salzer J.L. (2021). Activated microglia drive demyelination *via* CSF1R signaling.. Glia.

[93] Beckmann N., Giorgetti E., Neuhaus A., Zurbruegg S., Accart N., Smith P., Perdoux J., Perrot L., Nash M., Desrayaud S., Wipfli P., Frieauff W., Shimshek D.R. (2018). Brain region-specific enhancement of remyelination and prevention of demyelination by the CSF1R kinase inhibitor BLZ945.. Acta Neuropathol. Commun..

[94] Tahmasebi F., Pasbakhsh P., Mortezaee K., Madadi S., Barati S., Kashani I.R. (2019). Effect of the CSF1R inhibitor PLX3397 on remyelination of corpus callosum in a cuprizone‐induced demyelination mouse model.. J. Cell. Biochem..

[95] Tanabe S., Saitoh S., Miyajima H., Itokazu T., Yamashita T. (2019). Microglia suppress the secondary progression of autoimmune encephalomyelitis.. Glia.

[96] Kent S.A., Miron V.E. (2024). Microglia regulation of central nervous system myelin health and regeneration.. Nat. Rev. Immunol..

[97] Cantuti-Castelvetri L., Fitzner D., Bosch-Queralt M., Weil M.T., Su M., Sen P., Ruhwedel T., Mitkovski M., Trendelenburg G., Lütjohann D., Möbius W., Simons M. (2018). Defective cholesterol clearance limits remyelination in the aged central nervous system.. Science.

[98] Hametner S., Wimmer I., Haider L., Pfeifenbring S., Brück W., Lassmann H. (2013). Iron and neurodegeneration in the multiple sclerosis brain.. Ann. Neurol..

[99] Bagnato F., Hametner S., Yao B., van Gelderen P., Merkle H., Cantor F.K., Lassmann H., Duyn J.H. (2011). Tracking iron in multiple sclerosis: A combined imaging and histopathological study at 7 Tesla.. Brain.

[100] Mehta V., Pei W., Yang G., Li S., Swamy E., Boster A., Schmalbrock P., Pitt D. (2013). Iron is a sensitive biomarker for inflammation in multiple sclerosis lesions.. PLoS One.

[101] Gillen K.M., Mubarak M., Nguyen T.D., Pitt D. (2018). Significance and *in vivo* detection of iron-laden microglia in white matter multiple sclerosis lesions.. Front. Immunol..

[102] Neema M., Arora A., Healy B.C., Guss Z.D., Brass S.D., Duan Y., Buckle G.J., Glanz B.I., Stazzone L., Khoury S.J., Weiner H.L., Guttmann C.R.G., Bakshi R. (2009). Deep gray matter involvement on brain MRI scans is associated with clinical progression in multiple sclerosis.. J. Neuroimaging.

[103] Stüber C., Pitt D., Wang Y. (2016). Iron in multiple sclerosis and its noninvasive imaging with quantitative susceptibility mapping.. Int. J. Mol. Sci..

[104] Lloyd A.F., Miron V.E. (2019). The pro-remyelination properties of microglia in the central nervous system.. Nat. Rev. Neurol..

[105] Yeung M.S.Y., Djelloul M., Steiner E., Bernard S., Salehpour M., Possnert G., Brundin L., Frisén J. (2019). Dynamics of oligodendrocyte generation in multiple sclerosis.. Nature.

[106] Berghoff S.A., Spieth L., Saher G. (2022). Local cholesterol metabolism orchestrates remyelination.. Trends Neurosci..

[107] Berghoff S.A., Spieth L., Sun T., Hosang L., Schlaphoff L., Depp C., Düking T., Winchenbach J., Neuber J., Ewers D., Scholz P., van der Meer F., Cantuti-Castelvetri L., Sasmita A.O., Meschkat M., Ruhwedel T., Möbius W., Sankowski R., Prinz M., Huitinga I., Sereda M.W., Odoardi F., Ischebeck T., Simons M., Stadelmann-Nessler C., Edgar J.M., Nave K.A., Saher G. (2021). Microglia facilitate repair of demyelinated lesions *via* post-squalene sterol synthesis.. Nat. Neurosci..

[108] Cannon A.S., Nagarkatti P.S., Nagarkatti M. (2021). Targeting AhR as a novel therapeutic modality against inflammatory diseases.. Int. J. Mol. Sci..

[109] Neavin D.R., Liu D., Ray B., Weinshilboum R.M. (2018). The role of the aryl hydrocarbon receptor (AHR) in immune and inflammatory diseases.. Int. J. Mol. Sci..

[110] Wang Y., Liu T. (2015). Quantitative susceptibility mapping (QSM): Decoding MRI data for a tissue magnetic biomarker.. Magn. Reson. Med..

[111] Cignarella F., Filipello F., Bollman B., Cantoni C., Locca A., Mikesell R., Manis M., Ibrahim A., Deng L., Benitez B.A., Cruchaga C., Licastro D., Mihindukulasuriya K., Harari O., Buckland M., Holtzman D.M., Rosenthal A., Schwabe T., Tassi I., Piccio L. (2020). TREM2 activation on microglia promotes myelin debris clearance and remyelination in a model of multiple sclerosis.. Acta Neuropathol..

[112] Baaklini C.S., Ho M.F.S., Lange T., Hammond B.P., Panda S.P., Zirngibl M., Zia S., Himmelsbach K., Rana H., Phillips B., Antoszko D., Ibanga J., Lopez M., Lee K.V., Keough M.B., Caprariello A.V., Kerr B.J., Plemel J.R. (2023). Microglia promote remyelination independent of their role in clearing myelin debris.. Cell Rep..

[113] Groh J., Abdelwahab T., Kattimani Y., Hörner M., Loserth S., Gudi V., Adalbert R., Imdahl F., Saliba A.E., Coleman M., Stangel M., Simons M., Martini R. (2023). Microglia-mediated demyelination protects against CD8+ T cell-driven axon degeneration in mice carrying PLP defects.. Nat. Commun..

[114] Dal-Bianco A., Grabner G., Kronnerwetter C., Weber M., Höftberger R., Berger T., Auff E., Leutmezer F., Trattnig S., Lassmann H., Bagnato F., Hametner S. (2017). Slow expansion of multiple sclerosis iron rim lesions: Pathology and 7 T magnetic resonance imaging.. Acta Neuropathol..

[115] Absinta M., Maric D., Gharagozloo M., Garton T., Smith M.D., Jin J., Fitzgerald K.C., Song A., Liu P., Lin J.P., Wu T., Johnson K.R., McGavern D.B., Schafer D.P., Calabresi P.A., Reich D.S. (2021). A lymphocyte–microglia–astrocyte axis in chronic active multiple sclerosis.. Nature.

[116] Airas L., Rissanen E., Rinne J. (2017). Imaging of microglial activation in MS using PET: Research use and potential future clinical application.. Mult. Scler..

[117] Giannetti P., Politis M., Su P., Turkheimer F.E., Malik O., Keihaninejad S., Wu K., Waldman A., Reynolds R., Nicholas R., Piccini P. (2015). Increased PK11195-PET binding in normal-appearing white matter in clinically isolated syndrome.. Brain.

[118] Kaunzner U.W., Kang Y., Monohan E., Kothari P.J., Nealon N., Perumal J., Vartanian T., Kuceyeski A., Vallabhajosula S., Mozley P.D., Riley C.S., Newman S.M., Gauthier S.A. (2017). Reduction of PK11195 uptake observed in multiple sclerosis lesions after natalizumab initiation.. Mult. Scler. Relat. Disord..

[119] Rissanen E., Tuisku J., Vahlberg T., Sucksdorff M., Paavilainen T., Parkkola R., Rokka J., Gerhard A., Hinz R., Talbot P.S., Rinne J.O., Airas L. (2018). Microglial activation, white matter tract damage, and disability in MS.. Neurol. Neuroimmunol. Neuroinflamm..

[120] Khalil M., Teunissen C.E., Otto M., Piehl F., Sormani M.P., Gattringer T., Barro C., Kappos L., Comabella M., Fazekas F., Petzold A., Blennow K., Zetterberg H., Kuhle J. (2018). Neurofilaments as biomarkers in neurological disorders.. Nat. Rev. Neurol..

[121] Saraste M., Matilainen M., Vuorimaa A., Laaksonen S., Sucksdorff M., Leppert D., Kuhle J., Airas L. (2023). Association of serum neurofilament light with microglial activation in multiple sclerosis.. J. Neurol. Neurosurg. Psychiatry.

[122] Rommer P.S., Milo R., Han M.H., Satyanarayan S., Sellner J., Hauer L., Illes Z., Warnke C., Laurent S., Weber M.S., Zhang Y., Stuve O. (2019). Immunological aspects of approved MS therapeutics.. Front. Immunol..

[123] Healy L.M., Michell-Robinson M.A., Antel J.P. (2015). Regulation of human glia by multiple sclerosis disease modifying therapies.. Semin. Immunopathol..

[124] Simonsen C.S., Flemmen H.Ø., Broch L., Brunborg C., Berg-Hansen P., Moen S.M., Celius E.G. (2021). Early high efficacy treatment in multiple sclerosis is the best predictor of future disease activity over 1 and 2 years in a norwegian population-based registry.. Front. Neurol..

[125] Li D.K.B., Zhao G.J., Paty D.W. (2001). Randomized controlled trial of interferon-beta-1a in secondary progressive MS: MRI results.. Neurology.

[126] (2001). Secondary Progressive Efficacy Clinical Trial of Recombinant Interferon-Beta-1a in MS (SPECTRIMS) Study Group. Randomized controlled trial of interferon- beta-1a in secondary progressive MS: Clinical results.. Neurology.

[127] Krumbholz M., Faber H., Steinmeyer F., Hoffmann L.A., Kümpfel T., Pellkofer H., Derfuss T., Ionescu C., Starck M., Hafner C., Hohlfeld R., Meinl E. (2008). Interferon-β increases BAFF levels in multiple sclerosis: Implications for B cell autoimmunity.. Brain.

[128] Moore C.S., Cui Q.L., Warsi N.M., Durafourt B.A., Zorko N., Owen D.R., Antel J.P., Bar-Or A. (2015). Direct and indirect effects of immune and central nervous system-resident cells on human oligodendrocyte progenitor cell differentiation.. J. Immunol..

[129] Stoof T.J., Flier J., Sampat S., Nieboer C., Tensen C.P., Boorsma D.M. (2001). The antipsoriatic drug dimethylfumarate strongly suppresses chemokine production in human keratinocytes and peripheral blood mononuclear cells.. Br. J. Dermatol..

[130] Huang H., Taraboletti A., Shriver L.P. (2015). Dimethyl fumarate modulates antioxidant and lipid metabolism in oligodendrocytes.. Redox Biol..

[131] Greene S., Watanabe K., Braatz-Trulson J., Lou L. (1995). Inhibition of dihydroorotate dehydrogenase by the immunosuppressive agent leflunomide.. Biochem. Pharmacol..

[132] Bar-Or A., Pachner A., Menguy-Vacheron F., Kaplan J., Wiendl H. (2014). Teriflunomide and its mechanism of action in multiple sclerosis.. Drugs.

[133] Foster C.A., Howard L.M., Schweitzer A., Persohn E., Hiestand P.C., Balatoni B., Reuschel R., Beerli C., Schwartz M., Billich A. (2007). Brain penetration of the oral immunomodulatory drug FTY720 and its phosphorylation in the central nervous system during experimental autoimmune encephalomyelitis: Consequences for mode of action in multiple sclerosis.. J. Pharmacol. Exp. Ther..

[134] Kappos L., Radue E.W., O’Connor P., Polman C., Hohlfeld R., Calabresi P., Selmaj K., Agoropoulou C., Leyk M., Zhang-Auberson L., Burtin P. (2010). A placebo-controlled trial of oral fingolimod in relapsing multiple sclerosis.. N. Engl. J. Med..

[135] Lublin F., Miller D.H., Freedman M.S., Cree B.A.C., Wolinsky J.S., Weiner H., Lubetzki C., Hartung H.P., Montalban X., Uitdehaag B.M.J., Merschhemke M., Li B., Putzki N., Liu F.C., Häring D.A., Kappos L. (2016). Oral fingolimod in primary progressive multiple sclerosis (INFORMS): A phase 3, randomised, double-blind, placebo-controlled trial.. Lancet.

[136] Pham T.H.M., Okada T., Matloubian M., Lo C.G., Cyster J.G. (2008). S1P1 receptor signaling overrides retention mediated by G alpha i-coupled receptors to promote T cell egress.. Immunity.

[137] Dev K.K., Mullershausen F., Mattes H., Kuhn R.R., Bilbe G., Hoyer D., Mir A. (2008). Brain sphingosine-1-phosphate receptors: Implication for FTY720 in the treatment of multiple sclerosis.. Pharmacol. Ther..

[138] Noda H., Takeuchi H., Mizuno T., Suzumura A. (2013). Fingolimod phosphate promotes the neuroprotective effects of microglia.. J. Neuroimmunol..

[139] Kappos L., Li D.K.B., Stüve O., Hartung H.P., Freedman M.S., Hemmer B., Rieckmann P., Montalban X., Ziemssen T., Hunter B., Arnould S., Wallström E., Selmaj K. (2016). Safety and efficacy of siponimod (BAF312) in patients with relapsing-remitting multiple sclerosis.. JAMA Neurol..

[140] Gruchot J., Lein F., Lewen I., Reiche L., Weyers V., Petzsch P., Göttle P., Köhrer K., Hartung H.P., Küry P., Kremer D. (2022). Siponimod modulates the reaction of microglial cells to pro-inflammatory stimulation.. Int. J. Mol. Sci..

[141] Kollias G. (1999). The function of tumour necrosis factor and receptors in models of multi-organ inflammation, rheumatoid arthritis, multiple sclerosis and inflammatory bowel disease.. Ann. Rheum. Dis..

[142] Sharief M.K., Hentges R. (1991). Association between tumor necrosis factor-alpha and disease progression in patients with multiple sclerosis.. N. Engl. J. Med..

[143] Polman C.H., O’Connor P.W., Havrdova E., Hutchinson M., Kappos L., Miller D.H., Phillips J.T., Lublin F.D., Giovannoni G., Wajgt A., Toal M., Lynn F., Panzara M.A., Sandrock A.W. (2006). A randomized, placebo-controlled trial of natalizumab for relapsing multiple sclerosis.. N. Engl. J. Med..

[144] Ramos-Cejudo J., Oreja-Guevara C., Stark A.L. (2011). Rodriguez, de Antonio, L.; Chamorro, B.; Diez-Tejedor, E. Treatment with natalizumab in relapsing-remitting multiple sclerosis patients induces changes in inflammatory mechanism.. J. Clin. Immunol..

[145] Sucksdorff M., Tuisku J., Matilainen M., Vuorimaa A., Smith S., Keitilä J., Rokka J., Parkkola R., Nylund M., Rinne J., Rissanen E., Airas L. (2019). Natalizumab treatment reduces microglial activation in the white matter of the MS brain.. Neurol. Neuroimmunol. Neuroinflamm..

[146] Li R., Patterson K.R., Bar-Or A. (2018). Reassessing B cell contributions in multiple sclerosis.. Nat. Immunol..

[147] Hauser S.L., Kappos L., Arnold D.L., Bar-Or A., Brochet B., Naismith R.T., Traboulsee A., Wolinsky J.S., Belachew S., Koendgen H., Levesque V., Manfrini M., Model F., Hubeaux S., Mehta L., Montalban X. (2020). Five years of ocrelizumab in relapsing multiple sclerosis.. Neurology.

[148] Montalban X., Hauser S.L., Kappos L., Arnold D.L., Bar-Or A., Comi G., de Seze J., Giovannoni G., Hartung H.P., Hemmer B., Lublin F., Rammohan K.W., Selmaj K., Traboulsee A., Sauter A., Masterman D., Fontoura P., Belachew S., Garren H., Mairon N., Chin P., Wolinsky J.S. (2017). Ocrelizumab versus placebo in primary progressive multiple sclerosis.. N. Engl. J. Med..

[149] Hauser S.L., Bar-Or A., Cohen J.A., Comi G., Correale J., Coyle P.K., Cross A.H., de Seze J., Leppert D., Montalban X., Selmaj K., Wiendl H., Kerloeguen C., Willi R., Li B., Kakarieka A., Tomic D., Goodyear A., Pingili R., Häring D.A., Ramanathan K., Merschhemke M., Kappos L. (2020). Ofatumumab versus teriflunomide in multiple sclerosis.. N. Engl. J. Med..

[150] Steinman L., Fox E., Hartung H.P., Alvarez E., Qian P., Wray S., Robertson D., Huang D., Selmaj K., Wynn D., Cutter G., Mok K., Hsu Y., Xu Y., Weiss M.S., Bosco J.A., Power S.A., Lee L., Miskin H.P., Cree B.A.C. (2022). Ublituximab versus teriflunomide in relapsing multiple sclerosis.. N. Engl. J. Med..

[151] Bourdette D., Yadav V. (2008). B-cell depletion with rituximab in relapsing-remitting multiple sclerosis.. Curr. Neurol. Neurosci. Rep..

[152] Machado-Santos J., Saji E., Tröscher A.R., Paunovic M., Liblau R., Gabriely G., Bien C.G., Bauer J., Lassmann H. (2018). The compartmentalized inflammatory response in the multiple sclerosis brain is composed of tissue-resident CD8+ T lymphocytes and B cells.. Brain.

[153] Margoni M., Preziosa P., Filippi M., Rocca M.A. (2022). Anti-CD20 therapies for multiple sclerosis: Current status and future perspectives.. J. Neurol..

[154] Prosperini L., Annovazzi P., Boffa L., Buscarinu M.C., Gallo A., Matta M., Moiola L., Musu L., Perini P., Avolio C., Barcella V., Bianco A., Farina D., Ferraro E., Pontecorvo S., Granella F., Grimaldi L.M.E., Laroni A., Lus G., Patti F., Pucci E., Pasca M., Sarchielli P., Ghezzi A., Zaffaroni M., Baroncini D., Buttari F., Centonze D., Fornasiero A., Salvetti M., Docimo R., Signoriello E., Tedeschi G., Bertolotto A., Capobianco M., Comi G., Cocco E., Gallo P., Puthenparampil M., Grasso R., Di Francescantonio V., Rottoli M.R., Mirabella M., Lugaresi A., De Luca G., Di Ioia M., Di Tommaso V., Mancinelli L., Di Battista G., Francia A., Ruggieri S., Pozzilli C., Curti E., Tsantes E., Palmeri B., Lapicci C., Mancardi G.L., Uccelli A., Chisari C., D’Amico E., Cartechini E., Repice A.M., Magnani E., Massaccesi L., Calabresi P., Di Filippo M., Di Gregorio M. (2018). No evidence of disease activity (NEDA-3) and disability improvement after alemtuzumab treatment for multiple sclerosis: A 36-month real-world study.. J. Neurol..

[155] Jones J.L., Anderson J.M., Phuah C.L., Fox E.J., Selmaj K., Margolin D., Lake S.L., Palmer J., Thompson S.J., Wilkins A., Webber D.J., Compston D.A., Coles A.J. (2010). Improvement in disability after alemtuzumab treatment of multiple sclerosis is associated with neuroprotective autoimmunity.. Brain.

[156] Giovannoni G. (2017). Cladribine to treat relapsing forms of multiple sclerosis.. Neurotherapeutics.

[157] Jørgensen L.Ø., Hyrlov K.H., Elkjaer M.L., Weber A.B., Pedersen A.E., Svenningsen Å.F., Illes Z. (2020). Cladribine modifies functional properties of microglia.. Clin. Exp. Immunol..

[158] Krämer J., Bar-Or A., Turner T.J., Wiendl H. (2023). Bruton tyrosine kinase inhibitors for multiple sclerosis.. Nat. Rev. Neurol..

[159] Caldwell R.D., Qiu H., Askew B.C., Bender A.T., Brugger N., Camps M., Dhanabal M., Dutt V., Eichhorn T., Gardberg A.S., Goutopoulos A., Grenningloh R., Head J., Healey B., Hodous B.L., Huck B.R., Johnson T.L., Jones C., Jones R.C., Mochalkin I., Morandi F., Nguyen N., Meyring M., Potnick J.R., Santos D.C., Schmidt R., Sherer B., Shutes A., Urbahns K., Follis A.V., Wegener A.A., Zimmerli S.C., Liu-Bujalski L. (2019). Discovery of Evobrutinib: An oral, potent, and highly selective, covalent Bruton’s tyrosine kinase (BTK) inhibitor for the treatment of immunological diseases.. J. Med. Chem..

[160] Barboza A. (2024). It is time to rethink clinical trials on Bruton’s tyrosine kinase inhibitors in multiple sclerosis.. Mult. Scler. Relat. Disord..

